# Association between Depression, Antidepression Medications, and the Risk of Developing Type 2 Diabetes Mellitus: A Nationwide Population-Based Retrospective Cohort Study in Taiwan

**DOI:** 10.1155/2021/8857230

**Published:** 2021-01-07

**Authors:** Yi-Jen Fang, Tien-Yuan Wu, Jung-Nien Lai, Cheng-Li Lin, Ni Tien, Yun-Ping Lim

**Affiliations:** ^1^Research Center for Environmental Medicine, Kaohsiung Medical University, Kaohsiung, Taiwan; ^2^Ph.D. Program in Environmental and Occupational Medicine, College of Medicine, Kaohsiung Medical University and National Health Research Institutes, Taiwan; ^3^Graduate Institute of Clinical Medicine, Department of Environmental Health, Kaohsiung Medical University, Kaohsiung, Taiwan; ^4^National Institute of Environmental Health Sciences, National Health Research Institutes, Zhunan, Taiwan; ^5^Digestive Disease Center, Show Chwan Memorial Hospital, Changhua, Taiwan; ^6^Department of Pharmacy, Taichung Tzu Chi Hospital, Buddhist Tzu Chi Medical Foundation, Taichung, Taiwan; ^7^Department of Pharmacology, School of Medicine, Tzu Chi University, Hualien, Taiwan; ^8^Department of Chinese Medicine, China Medical University Hospital, Taichung, Taiwan; ^9^School of Chinese Medicine, College of Chinese Medicine, China Medical University, Taichung, Taiwan; ^10^Management Office for Health Data, China Medical University Hospital, Taichung, Taiwan; ^11^Department of Laboratory Medicine, China Medical University Hospital, Taichung, Taiwan; ^12^Department of Medical Laboratory Science and Biotechnology, China Medical University, Taichung, Taiwan; ^13^Department of Pharmacy, College of Pharmacy, China Medical University, Taichung, Taiwan; ^14^Department of Internal Medicine, China Medical University Hospital, Taichung, Taiwan; ^15^Department of Medical Research, China Medical University Hospital, Taichung, Taiwan

## Abstract

The relationship between depression, antidepressant medications (ADMs), and the risk of subsequent type 2 diabetes mellitus (T2DM) development remains controversial. Thus, we investigated this aspect by a population-based retrospective cohort study using the Longitudinal Health Insurance Database 2000 available in Taiwan. This large, observational study included 46,201 patients with depression and a 1 : 1 age- and sex-matched nondepression cohort enrolled between January 1, 2000, and December 31, 2013, and the newly diagnosed T2DM incidence rates were determined. We estimated the effects of depression on T2DM and the cumulative incidence curves by Cox proportional regression hazard models and Kaplan-Meier methods, respectively. We found that 47.97% of the patients with depression did not receive ADM. Among patients with depression who received ADM, 29.71%, 6.29%, 0.05%, 9.65%, and 6.32% received selective serotonin reuptake inhibitors (SSRIs), tricyclic antidepressants (TCAs), monoamine oxidase inhibitors (MAOIs), heterocyclic antidepressants, and other medications, respectively. Patients without ADM treatment had a 39% higher risk of developing T2DM. However, those who received ADM treatment had a significantly lower risk of T2DM development in every treatment category. Depressive disorder treated with ADMs, especially with long-term use, was associated with an 11–48% decrease in the risk of T2DM in all ADM groups; however, heterocyclic antidepressant treatment for shorter periods (<80 days) was not significantly associated with a decreased risk of T2DM. The incidence of T2DM in Taiwan was found to be associated with an *a priori* history of depression and was inversely correlated with ADM treatment.

## 1. Introduction

The global prevalence of age-standardized type 2 diabetes mellitus (T2DM) was predicted to be 9.3% (463 million people) in 2019, and it is projected to rise to 10.2% (578 million) and 10.9% (700 million) by 2030 and 2045, respectively [1]. Diabetes can lead to several complications, including damage to the eyes, heart, kidneys, nerves, and blood vessels, and approximately 50% of DM patients die of cardiovascular disease [1]. The World Health Organization (WHO) warns that DM will be the seventh leading cause of death in 2030. The risk factors associated with T2DM are multiplex but largely comprise rapid increases in overweight, high body mass index, less physical activity, sedentary lifestyles, and high frequencies of high fat intake. In addition, a few studies have also demonstrated depression to be a risk factor or to be associated with the development of T2DM [2, 3]. The relationship between depression and the onset of DM has been investigated earlier but has revealed inconsistent findings. Some researchers report that depression is associated with an increased risk of developing T2DM, whereas other studies have found no significant association. According to one study, people with depression have a 41% higher risk for developing DM and a 32% higher risk for developing T2DM compared to those without depression [4]. However, the exact mechanisms underlying this correlation are still uncertain and need further investigation.

Depression is one of the most common psychiatric disorders. Patients with depression experience a dysphoric state of mind, loss of intrigue and joy, aggravated hunger and lethargy, and changes in vitality levels. Accordingly, diminished self-care practices are frequently connected with depression, for example, diminished adherence to prescribed medication and proper diet, as well as a lack of physical activity [5]. In a 38-state overview in the United States, the general predominance of current burdensome side effects was found to be 8.7%, with a 15.7% lifetime diagnosis of depression as determined by a specialist or healthcare service provider [6, 7].

The use of antidepressant medication (ADM) has significantly increased in the past decade in many countries, such as USA and Taiwan [8, 9]. It has become one of the most commonly prescribed classes of medication in outpatient medical practice [9, 10]. These medications have been reported to have various adverse consequences, such as weight gain [11] and impaired glucose homeostasis [12, 13]. Several reports have also linked ADM use with the risk of T2DM; however, the results remain limited and inconsistent [14–19]. In addition, depression is associated with extensive morbidity and mortality [20]. It is also reported to associate with a number of chronic diseases, such as acute myocardial infarction, chronic obstructive pulmonary disease (COPD), stroke, and coronary artery disease (CAD) [21, 22]. The beginning of depression may bring about increased weight gain (however, this may be because of the altered eating behavior or the side effects of treatment with antidepressants) and diminished self-care, for example, lack of physical exercise. Additionally, individuals with depression have a higher tendency to consume liquor and smoke cigarettes compared to people without depression [3]. These practices can conceivably accelerate the onset of T2DM. However, the literature evaluating depression has not provided consistent and clear associations among these factors.

T2DM and depression are significant comorbid conditions, and comorbid depression is related with more severe outcomes in individuals with T2DM. It is not entirely established, however, whether those with a history of depression are somehow predisposed to developing T2DM. The potential causal relationship has important implications for understanding the mechanisms, whereby depression might predispose individuals diagnosed with depression to T2DM. Because T2DM affects large populations and the quality of life of patients and induces several reversible or irreversible complications, verifying the association between depression, antidepressants, and the subsequent development of T2DM is crucial. Few epidemiological studies have investigated this relationship, especially in Asian populations. Thus, to assess the possible relationship of depression, antidepressant use, and the risk related to the development of T2DM, we conducted a population-based retrospective cohort study by using data from a large administrative dataset from the Taiwan National Health Insurance Research Database (NHIRD).

## 2. Materials and Methods

### 2.1. Data Source

The NHIRD is derived from the government-operated single-payer National Health Insurance (NHI) program. NHI program is a universal public program in Taiwan that was implemented in 1995 and covers 99.5% of the 23 million residents in Taiwan. This population-based retrospective cohort study was conducted using the Longitudinal Health Insurance Database (LHID2000), which comprises detailed healthcare data of one million randomly selected and enrolled representative subjects from the NHIRD in 2000. Diagnostic codes were based on the International Classification of Diseases, 9th Revision, Clinical Modification (ICD-9-CM). This study was approved by the Research Ethics Committee at China Medical University Hospital (CMUH104-REC2-115-CR-4), which waived the requirement for informed consent because the personal information was anonymized and de-identified for research purposes.

### 2.2. Study Participants

We identified subjects with newly diagnosed depression (ICD-9-CM codes 296.2, 296.3, 296.82, 300.4, and 311, but excluding populations with the diagnosis codes 296.0, 296.1, 296.4, 296.5, 296.6, 296.7, 296.8, 296.80, and 296.89) from January 1, 2000, to December 31, 2013, as the depression cohort. The date of first diagnosis of depression was set as the index date. Subjects with a history of T2DM before the index date were excluded from the study. Subjects without a history of depression during the study period were randomly selected from the same database as the comparison cohort. For each subject in the depression cohort, one subject from the comparison cohort was selected through frequency matching according to age (every 5-year span), sex, and index year under the same exclusion criteria between the two cohorts.

### 2.3. Major Outcomes, Comorbidites, and Medications

The primary outcome was newly diagnosed T2DM (ICD-9-CM code 250). The follow-up began from the index date until the date of T2DM diagnosis, withdrawal from the NHI program, or the end of 2013, whichever came first. The T2DM group included two cohorts, with ≥1 admission or ≥2 ambulatory care visits for T2DM (ICD-9-CM codes 250. ×0 or 250. ×2) between 2000 and 2013 and excluded diagnoses of T1DM (ICD-9-CM codes 250. ×1 or 250. ×3) or gestational diabetes (ICD-9-CM code 648. ××) during 2000 to 2013. Several comorbidities and medications were considered as potential confounders in this study, namely, hypertension (ICD-9-CM codes 401–405); stroke (ICD-9-CM codes 430–438); hyperlipidemia (ICD-9-CM code 272); COPD (ICD-9-CM codes 491, 492, and 496); alcohol-related illness (ICD-9-CM codes 291, 303, 305, 571.0, 571.1, 571.2, 571.3, 790.3, A215, and V11.3); sleep disorder (ICD-9-CM codes 307, 327, and 780.5) [23, 24]; asthma (ICD-9-CM code 493) [25]; anxiety (ICD-9-CM code 300) [26]; obesity (ICD-9-CM code 278.00); nonalcoholic steatohepatitis (NASH) (ICD-9-CM code 571.8); and the use of medications such as steroids, SSRIs, TCAs, MAOIs, heterocyclic antidepressants, and other antidepressants. We excluded users of more than one antidepressant to ensure defined exposure categories.

### 2.4. Statistical Analysis

The chi-square test and two-sample *t* test were used to examine the difference for categorical variables and continuous variables between patients with and without depression, respectively. We measured the cumulative incidence curves between the depression and comparison cohorts using the Kaplan-Meier method and tested the difference with the log-rank test. The incidence rate of T2DM was calculated per 1,000 person-years. The unadjusted and multivariable-adjusted hazard ratios (HR and aHR) with corresponding 95% confidence intervals (CIs) for T2DM risk between depression and comparison cohorts were analyzed using the Cox proportional hazard regression model. The association between T2DM and ADMs was also assessed. In addition, we divided each antidepressant type into two groups according to the cumulative duration of drug use to evaluate the effect of ADMs on the risk of T2DM development. All statistical analyses were performed using SAS software, version 9.4 (SAS Institute, Inc.), and the significance level was set at *p* < 0.05.

## 3. Results

Table 1 lists the demographic characteristics and comorbidities of the depression cohort and the comparison cohort, and medication use is presented for the depression cohort. No significant differences in sex and age were observed between the depression and comparison cohorts. The depression cohort presented significantly higher proportions of hypertension, stroke, hyperlipidemia, COPD, CAD, sleep disorder, asthma, and anxiety (*p* < 0.001) than those in the comparison cohort. Among patients with depression, 29.71%, 6.29%, and 9.65% received treatment with SSRIs, TCAs, and heterocyclic antidepressants, respectively.

**Figure 1:**
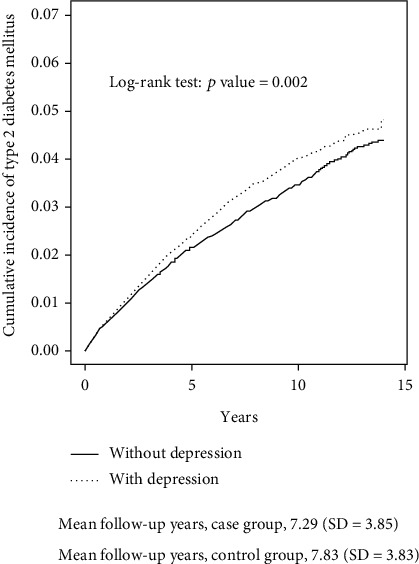
Cummulative incidence of type 2 diabetes mellitus (T2DM) compared between with and without depression (Kaplan-Meier method).

The mean duration of follow-up years in the depression cohort and the comparison cohort were 7.29 ± 3.85 and 7.83 ± 3.83 years, respectively. The cumulative risk of developing T2DM was calculated by the Kaplan-Meier method (Figure 1). The depression cohort had a significantly higher rate of developing T2DM than that of the comparison cohort (log-rank test, *p* = 0.002).

Table 2 shows the Cox regression analysis of the risk of T2DM between the two cohorts stratified by sex, age, and comorbidity. The overall incidence of T2DM was higher in the depression cohort than in the comparison cohort (4.21 vs. 3.68 per 1,000 person-years). In multivariate Cox regression model analysis, patients in the depression cohort showed a significantly higher risk of T2DM, with an aHR of 1.17 (95%CI = 1.11–1.23). In the stratified analysis, the risk of T2DM in the depression cohort was higher than that in the comparison cohort among female patients (aHR = 1.25, 95%CI = 1.17–1.33). Patients with depression in all age groups were independently associated with a higher risk of T2DM than in the comparison cohort (aHR = 1.14, 95%CI = 1.05–1.23 in the ≤49 years age group; aHR = 1.15, 95%CI = 1.03–1.28 in the 50–64 years age group; aHR = 1.15, 95%CI = 1.03–1.29 in the ≥65 years age group). As for stratification by comorbidity, no significant association between depression and T2DM was observed among patients with and without comorbidity.

Table 3 presents the effect of different antidepressant treatments on the risk of developing T2DM. Compared with nondepression control subjects, the risk of T2DM was significantly lower for patients using SSRIs (aHR = 0.67, 95%CI = 0.62–0.72), TCAs (aHR = 0.63, 95%CI = 0.56–0.70), heterocyclic antidepressants (aHR = 0.90, 95%CI = 0.82–0.98), and other antidepressants (aHR = 0.69, 95%CI = 0.59–0.80).

Table 4 shows that the risk of T2DM decreased with the cumulative duration of use of a specific type of antidepressant. The risk of T2DM was significantly lower for SSRI users for both <120 days (aHR = 0.66, 95%CI = 0.62–0.71) and ≥120 days (aHR = 0.52, 95%CI = 0.49–0.56) compared with SSRI nonusers. The risk of T2DM was significantly lower for TCA users for both <35 days (aHR = 0.78, 95%CI = 0.72–0.83) and ≥35 days (aHR = 0.73, 95%CI = 0.68–0.78) compared with TCAs nonusers. The risk of T2DM was significantly lower for heterocyclic users for ≥80 days (aHR = 0.89, 95%CI = 0.84–0.96) compared with heterocyclic nonusers (Table 4).

## 4. Discussion

In this comprehensive nationwide population-based cohort study, we investigated the possible association of depression and the development of T2DM under ADM therapy. We found that (1) the overall association of depression and T2DM was 17% higher in the depression cohort than in the comparison cohort; (2) patients with depression who were not treated with ADMs had a 39% higher risk of T2DM development compared to the comparison cohort; and (3) after treatment with ADMs, the incidence of T2DM was lower in patients with depression than in those without ADM treatment (all aHR < 0.90). This single cohort study included a large number of participants and had a long study duration, and the outcome was adjusted for most confounding factors.

The relationship between depression and T2DM is of particular interest because both chronic diseases are major contributors to the global burden of pharmaceutical use. Several epidemiological studies have investigated a bidirectional association between depression and T2DM, implying that depression increases the risk of developing T2DM, and in turn, T2DM is also associated with an increased tendency for developing depression [27, 28]. Therefore, improving the incidence of T2DM in patients with depression might be more important than improving it in the general population.

Our results are supported by recently published data from a longitudinal investigation between depression and the onset of T2DM. Some report that depression is associated with an increased risk of developing T2DM, whereas other studies have not found a significant association. A meta-analysis by Yu et al. showed that people with depression have a 41% increased risk of developing DM and a 32% increased risk of developing T2DM [4]. In our results, the overall incidence of T2DM was 17% higher in patients with depression, with or without ADMs, than the comparison cohort, consistent with this meta-analysis report. However, we also found a relationship between ADM use in patients with depression with a decreased risk of T2DM.

Several meta-analyses have reported on depression and its relationship with T2DM [27, 29–31]. Cosgrove et al. [30] found that the relative risk (RR) for T2DM was 1.25 (95% CI, 1.02–1.48), and Knol et al. [29] also believed that depression could increase the risk of T2DM; the combined RR was 1.37 (1.14–1.63). A study conducted by Mezuk et al. [27] revealed that the RR for incident diabetes associated with baseline depression was 1.60 (1.37–1.88), and another publication [31] presented the RR for diabetes as 1.38 (95% CI, 1.23–1.55). Our results show that the aHR is 1.17 (95%CI = 1.11–1.23), similar with previous findings. Observational studies have produced conflicting data on the risk of T2DM in ADM users [14–19]. For ADM use and the risk of T2DM, however, the results remain limited and inconsistent owing to limitations such as case-control studies, small size, or being restricted to high-risk populations.

Knol et al. [14] did not find an increased risk of diabetes in individuals using ADMs by using prescription data from the PHARMO database. Some other studies suggest an association between ADM use and T2DM. One study revealed that the long-term use of ADMs in at least moderate daily doses was associated with an increased risk of T2DM in both TCA and SSRI groups [15]. An analysis of spontaneous reports of adverse drug reactions recorded in the WHO's Adverse Drug Reaction Database showed increased risks of hyperglycemia and hypoglycemia associated with the use of ADMs and significantly increased risk of hyperglycemia with ADM use for >1 year [32].

Whether ADMs elevate or reduce T2DM risk remains controversial. In a study of three cohorts conducted over 12–16 years and comprising over 160,000 US participants, the use of SSRIs and TCAs was found to be related with a higher risk of T2DM (pooled HR = 1.10 and 1.26, respectively) [33]. In the Diabetes Prevention Program (DPP) study, Rubin et al. [16] found that SSRI use was related with over a twofold higher risk of T2DM among patients with impaired glucose tolerance. Recently, two large case-control studies utilizing clinical record information databases in the UK [15] and Finland [17] found an increased risk of T2DM related with long-term ADM (SSRIs and TCAs) use of moderate and additionally high daily doses for depression treatment, and the association was independent of the severity of depression. A cohort study in over 1,000 Australians found that ADM use was related with an 80% increased hazard for T2DM, although this was not significant [18]. In a Spanish population of individuals aged ≥55 years, the HR for ADM intake was 1.26 [19]. Treatment with ADMs was associated with an increased risk of T2DM. However, from Pan's study, information on ADM use was self-reported, and they could not assess the association between specific associated drugs, doses, and duration of use with diabetes risk. Furthermore, they lacked clinical data on participants' depression history, such as their severity and chronicity [28]. The present study results revealed that patients with depression who did not receive ADMs had an increased risk of developing T2DM. However, patients with depression who received ADMs, especially those treated with SSRIs, had the most significantly reduced risk of developing T2DM. However, whether ADM use is a causal risk factor for T2DM or acts as a marker of depression seriousness/chronicity remains to be ascertained. In the present study, exposure to short-term or long-term treatment or each ADM category was associated with decreased T2DM risk. Potential diabetes risk is currently not considered in clinical guidelines for treating depression. We observed a substantially decreased risk of T2DM associated with continuing ADM use. Findings from this study suggest that the use of SSRIs and TCAs may be associated with a reduced risk of T2DM in patients with depression after adjustment for several confounding factors associated with diabetes risk and other covariates. The reduction in T2DM risk was not statistically significant for heterocyclic ADM use for <80 days treatment, but a significantly reduced risk of 10% was found for ≥80 days treatment, indicating that the reducing effect of T2DM incidence might be very weak, given the observational study setting.

It is likely that there is a direct association between depression and the subsequent development of T2DM. Several possibilities have been proposed, listed as follows. Depression may directly lead to T2DM by influencing the activity of several neurotransmitters, such as acetylcholine, dopamine, *γ*-aminobutyric acid, norepinephrine, and serotonin [34], thereby increasing inflammation-related markers that may lead to the pathogenesis of T2DM. A major depressive episode may disturb the hypothalamo-pituitary-adrenal axis by increasing corticotropin-releasing hormone levels and thus increasing plasma corticosteroid hormone levels. Therefore, depression and T2DM might determine central adiposity, because it is proposed that depression causes a more central distribution of fat, which in turn causes T2DM [35]. Hypothalamic anomalies increase abdominal circumference, free fatty acid concentration, heart rate, cardiac yield, and renin discharge, along with excess cortisol and androgen generation. Brain-derived neurotrophic factor (BDNF) might act as a neurotrophin, immunotrophin, epitheliotrophin, and metabotrophin [36]. Animal tests have demonstrated decreased BDNF to be related with depressive states and eating behavior, and clinical studies have shown that BDNF administration can reverse these states [37]. Thus, BDNF may play a vital role in the pathogenesis of depression and should be included within the restorative impacts of antidepressants. Serum BDNF concentrations were diminished in patients with depression [38, 39]. Karege et al. have reported that BDNF is closely related with T2DM and has a major impact on the pathogenesis of weight and T2DM via alteration of the secretion and action of insulin, leptin, ghrelin, neurotransmitters/neuropeptides, and proinflammatory cytokines related with vitality homeostasis [38]. Thus, BDNF may represent a link between depression and T2DM. These findings appear to be consistent with our results, wherein patients with depression without ADMs showed an increased risk of T2DM development; however, with ADMs, this effect could be reversed, although we did not detect BDNF directly from the patients' serum. Thus, ADMs that act on the hypothalamo-pituitary-adrenal axis may rescue patients from the risk of T2DM. In addition, the development of T2DM in patients with depression might be related to depressive behavior (inactivity, tendency to have body weight changes, and less likelihood of engaging in healthy behavior such as exercise and taking a healthy diet), which increases the risk of developing diabetes and obesity. We hypothesize that when depressive patients receive ADMs, they might have a better quality of life and a lower risk of T2DM. Future studies in which endocrine levels are monitored in patients with depression are necessary to confirm this hypothesis.

We report that the risk of T2DM in patients with depression was higher in female patients than in male patients. Several studies have arrived at similar conclusions. One group aimed to assess the prevalence of T2DM-related distress, glycemic control, and association with depression and their related factors in Jazan, Saudi Arabia. After adjusting for covariates, they found that female sex, age of <45 years, physical inactivity, DM duration of <5 years, and smoking were significantly associated with DM-related distress and depression [40]. Another study investigated the prevalence of generalized anxiety disorder (GAD) in Taiwanese patients with T2DM. During the investigation period of 2000 to 2010, the prevalence of GAD was significantly greater in the T2DM patients than in the general control cohort, while the increase in GAD was higher in the general control cohort (from 0.25% to 0.63%) than among T2DM patients (from 0.81% to 1.03%). Huang et al. [41] showed that in T2DM patients, GAD was associated with female sex. Another study investigated the prevalence of anxiety disorder (AD) in Taiwanese patients with T2DM. A high prevalence of AD in patients with T2DM was associated with an age of >30 years; female sex; living in the northern region of Taiwan; comorbidities of congestive heart failure, peripheral vascular disease, CVD, and depression disorder; and a Charlson participant comorbidity index of ≥1 [42].

Further investigations using more detailed data on dosage and length of treatment and other clinical factors are warranted to confirm our findings. Moreover, mechanistic studies are required to clarify the impact of antidepressants on glucose resistance and carbohydrate digestion. Recent conclusive proof on this relationship has been obtained, and patients with depression are exhorted to follow their treatment regimen with cautious consideration of their body weight and blood glucose level [43]. Besides, the effect of certain types of ADM should be interpreted carefully because the reasons for prescribing particular ADMs may vary. The predominance of SSRI use was the highest in our cohorts (29.37%), which was coincident with SSRIs being used as the first-line treatment for depression amid follow-up.

Why these ADMs should be associated with a reduced risk of depression in people with T2DM is not clear. This study confirms that the management of depression remains poor. Only 48.66% of patients with depression received ADMs. Therefore, future studies need to be more specific on types of ADM. Further prospective, interventional, and properly powered studies are required in patients with features of depression and/or T2DM to investigate the effects of ADMs, including SSRIs, TCAs, heterocyclics, and other medications, on depression and the possible role of ADMS in the etiopathogenesis of insulin resistance and diabetes. This study suggests a possible role of depression and type- and duration-related associations between ADM use and reduced risk of T2DM in adults with depression. ADMs might have a role in not only treating depression but also reducing the likelihood of developing T2DM. As quality of life and life expectancy improve for patients with depression, the use of tolerable therapies that have multiple beneficial effects should be promoted.

The strengths of this study are as follows: (1) this was a population-based cohort study based on information from NHIRD records; (2) it was based on ICD-9-CM codes, which are more accurate and reliable compared to self-reported information of the drugs taken by the patients; (3) the study had a long-term follow-up period; (4) data on the onset of diverse conditions and comorbidities related to T2DM in patients and drugs consumed were obtained; and (5) Cox proportional regression hazard models minimized the confounding factors.

However, this study had the following limitations as well: (1) a few unmeasured confounding factors might have interfered with the outcomes of the study. Some confounding components affecting the pathophysiology of T2DM, such as patients' personal information, including liquor intake, smoking status, body mass index (BMI), natural presentation, working status, and family history of T2DM, were inaccessible in NHIRD. However, in a meta-analysis, Khapre et al. [44] obtained data from the Cochrane database and carried out a systematic investigation in a parallel randomized clinical trial with a control group and patients receiving ADMs; the outcome measures were HbA1c, fasting blood glucose, body weight, BMI, and treatment adherence. They found that of 394 studies, 6 studies fulfilled their setting criteria, which were selected for further analysis. Using mean difference (MD), their meta-analysis revealed that glycemic control was more favorable in the ADM-treated DM population than in the control group (MD = −0.32%; 95%CI = −0.57–0.08). They found that weight and BMI did not show any significant MD between these two populations. Another group investigated the impact of BMI on serum concentrations of several kinds of ADMs and considered confounding parameters such as age, sex, and smoking habit [45, 46]. They found no association between BMI and serum concentrations of the tested ADMs. Thus, we believe that BMI and weight changes may not impact the interpretation of our present results. (2) The NHIRD claims database was initially built for charging purposes. Hence, some information was anonymized, and we could not obtain individual data of the patients after reaching out to them directly. (3) No information on dietary intake was accessible in this database. (4) Because information from certain research facilities was not included in NHIRD, we could not make deductions based on variables such as blood glucose and glycated hemoglobin (HbA1c). Thus, our case definition was based on physician-recorded diagnoses according to patients' blood glucose status after ADM treatment indirectly. In our study, both depression and diabetes were accurately analyzed and coded (ICD-9-CM codes) by specialists according to the standard symptomatic criteria, considering normal side effects and signs, research facility information, and imaging findings. Therefore, confounding effects affecting comorbidities were minimized. The severity and length of depression may influence T2DM. In Taiwan, major depression tends to be underdiagnosed or at least undercharted, similar to other countries. Nonetheless, unlike other countries, in Taiwan, ADMs are not easily prescribed by psychiatrists unless the patients are diagnosed with major depression or related psychotic disorders. Therefore, the data on ADMs in our study are reliable and closely associated with the diagnosis of major depression. To conduct a prospective study or randomized controlled trial investigating the relationship between depression, ADMs, and T2DM in the future, it is necessary to obtain more information from other databases or questionnaires. An essential part of optimizing care for patients with depression is likely to be physician education to improve knowledge and promote awareness about depression and associated risk factors.

## 5. Conclusion

The study results indicated that T2DM development was associated with a history of depression, while ADM use was associated with a reduced risk of the subsequent development of T2DM. However, in the future, large, population-based studies or large-scale randomized clinical trials will be required to confirm our observation that these ADMs play a role in preventing the development of T2DM before any definitive conclusions can be drawn.

## Figures and Tables

**Table 1:** Demographic characteristics, comorbidity, and medication in patient with and without depression.

Variable	Depression		*p* value
	No	Yes	
	*N* = 46201	*N* = 46201	
Sex	*N* (%)	*N* (%)	0.99
Female	28278 (61.2)	28278 (61.2)	
Male	17923 (38.8)	17923 (38.8)	
Age, mean (SD)	46.2 (17.5)	46.7 (17.3)	0.001
Stratify age			0.99
≤49	27890 (60.4)	27890 (60.4)	
50-64	10388 (22.5)	10388 (22.5)	
65+	7923 (17.2)	7923 (17.2)	
Comorbidity			
Hypertension	9321 (20.2)	13549 (29.3)	<0.001
Stroke	822 (1.78)	2058 (4.45)	<0.001
Hyperlipidemia	6737 (14.6)	10802 (23.4)	<0.001
COPD	3981 (8.62)	6887 (14.9)	<0.001
CAD	5086 (11.0)	9430 (20.4)	<0.001
Sleep disorder	7463 (16.2)	26067 (56.4)	<0.001
Asthma	2947 (6.38)	5000 (10.8)	<0.001
Anxiety	4989 (10.8)	36718 (79.5)	<0.001
Obesity	285 (0.62)	491 (1.06)	<0.001
NASH	484 (1.05)	1084 (2.35)	<0.001
Medication			
Steroids	30981 (67.1)	37058 (80.2)	<0.001
SSRIs		13728 (29.7)	
Tricyclics		2908 (6.29)	
MAOIs		23 (0.05)	
Heterocyclic antidepressants		4457 (9.65)	
Others		2921 (6.32)	

Chi-square test; ^#^two sample *t* test.

**Table 2:** Comparison of incidence and hazard ratio of type 2 diabetes mellitus (T2DM) stratified by sex, age, and comorbidity between with and without depression.

Variable	Depression						Crude HR (95% CI)	Adjusted HR^†^ (95% CI)
	No			Yes				
	Event	PY	Rate^#^	Event	PY	Rate^#^		
All	1333	361778	3.68	1418	336639	4.21	1.14 (1.10, 1.19)^∗∗∗^	1.17 (1.11, 1.23)^∗∗∗^
Sex								
Female	759	225625	3.36	842	211927	3.97	1.18 (1.12, 1.24)^∗∗∗^	1.25 (1.17, 1.33)^∗∗∗^
Male	574	136153	4.22	576	124712	4.62	1.10 (1.03, 1.17)^∗^	1.08 (0.99, 1.17)
Stratify age								
≤49	333	231563	1.44	452	217255	2.08	1.45 (1.37, 1.53)^∗∗∗^	1.14 (1.05, 1.23)^∗∗^
50-64	504	79237	6.36	486	73175	6.64	1.04 (0.97, 1.13)	1.15 (1.03, 1.28)^∗^
65+	496	50977	9.73	480	46209	10.4	1.07 (0.98, 1.17)	1.15 (1.03, 1.29)^∗^
Comorbidity‡								
No	443	224659	1.97	43	31472	1.37	0.69 (0.61, 0.79)^∗∗∗^	0.96 (0.86, 1.09)
Yes	890	137119	6.49	1375	305166	4.51	0.69 (0.66, 0.73)^∗∗∗^	0.93 (0.89, 0.98)^∗∗^

Rate^#^: incidence rate, per 1,000 person-years; Crude HR: crude hazard ratio. Adjusted HR^†^: multivariable analysis including age, sex, and comorbidities of hypertension, stroke, hyperlipidemia, COPD, CAD, sleep disorder, asthma, anxiety, obesity, and NASH, and steroids. Comorbidity‡: patients with any one of the comorbidities hypertension, stroke, hyperlipidemia, COPD, CAD, sleep disorder, asthma, anxiety, obesity, and NASH were classified as the comorbidity group. ^∗^*p* < 0.05, ^∗∗^*p* < 0.01, ^∗∗∗^*p* < 0.001.

**Table 3:** Incidence, crude, and adjusted hazard ratio of type 2 diabetes mellitus (T2DM) compared among depression patients with and without antidepressant treatment compared to nondepression controls.

Variables	*N*	Event	PY	Rate^#^	Crude HR (95% CI)	Adjusted HR^†^ (95% CI)	Adjusted HR^†^ (95% CI)
Nondepression controls	46201	1333	361778	3.68	1 (reference)	1 (reference)	
Depression without antidepressant treatment	22164	871	152086	5.73	1.55 (1.49, 1.63)^∗∗∗^	1.39 (1.31, 1.47)^∗∗∗^	1(reference)
Depression with antidepressant treatment							
SSRIs	13728	261	101473	2.57	0.70 (0.65, 0.75)^∗∗∗^	0.94 (0.87, 1.01)	0.67 (0.62, 0.72)^∗∗∗^
Tricyclics	2908	91	26468	3.44	0.93 (0.83, 1.04)	0.86 (0.77, 0.96)^∗^	0.63 (0.56, 0.70)^∗∗∗^
Heterocyclic antidepressants	4457	146	36343	4.02	1.09 (1.00, 1.19)	1.25 (1.14,1.38)^∗∗∗^	0.90 (0.82, 0.98)^∗^
MAOIs	23	0	214	0.00	—	—	—
Others	2921	49	20054	2.44	0.66 (0.57, 0.77)^∗∗∗^	0.98 (0.84, 1.13)	0.69 (0.59, 0.80)^∗∗∗^

Rate^#^: incidence rate, per 1,000 person-years; Crude HR: crude hazard ratio. Adjusted HR^†^: multivariable analysis including age, sex, and comorbidities of hypertension, stroke, hyperlipidemia, COPD, CAD, sleep disorder, asthma, anxiety, obesity, and NASH, and steroids. ^∗^*p* < 0.05, ^∗∗^*p* < 0.01, ^∗∗∗^*p* < 0.001.

**Table 4:** Hazard ratio and 95% confidence intervals of type 2 diabetes mellitus (T2DM) associated with cumulative the number of days of SSRIs exposure, tricyclics exposure, and heterocyclic antidepressants exposure among depression patients.

	Event/N	Crude HR^∗^	(95% CI)	Adjusted HR^†^	(95% CI)
Nonuse of SSRIs	687/14884	1	(Reference)	1	(Reference)
SSRIs^#^					
<120 days	387/15807	0.53	(0.50, 0.57)^∗∗∗^	0.66	(0.62, 0.71)^∗∗∗^
≥120 days	344/15510	0.46	(0.43, 0.49)^∗∗∗^	0.52	(0.49, 0.56)^∗∗∗^
Nonuse of TCAs	771/26787	1	(Reference)	1	(Reference)
TCAs^#^					
<35 days	285/9561	0.94	(0.87, 1.01)	0.78	(0.72, 0.83)^∗∗∗^
≥35 days	362/19850	1.11	(1.05, 1.20)^∗∗^	0.73	(0.68, 0.78)^∗∗∗^
Nonuse of heterocyclic antidepressants	726/24557	1	(Reference)	1	(Reference)
Heterocyclic antidepressants^#^					
<80 days	352/11034	1.01	(0.94, 1.08)	0.98	(0.92, 1.04)
≥80 days	340/10610	0.97	(0.91, 1.04)	0.89	(0.84, 0.96)^∗∗^

^#^The annual mean the number of days is partitioned in to 2 segments by median. Crude HR^∗^: relative hazard ratio. Adjusted HR^†^: multivariable analysis including age, sex, and comorbidities of hypertension, stroke, hyperlipidemia, COPD, CAD, sleep disorder, asthma, anxiety, obesity, and NASH, and steroids. ^∗^*p* < 0.05, ^∗∗^*p* < 0.01, ^∗∗∗^*p* < 0.001.

## Data Availability

The data used to support the findings of this study are included within the article.
